# Humoral responses to the SARS-CoV-2 spike and receptor binding domain in context of pre-existing immunity confer broad sarbecovirus neutralization

**DOI:** 10.3389/fimmu.2022.902260

**Published:** 2022-08-04

**Authors:** Blake M. Hauser, Maya Sangesland, Evan C. Lam, Jared Feldman, Alejandro B. Balazs, Daniel Lingwood, Aaron G. Schmidt

**Affiliations:** ^1^ Ragon Institute of Massachusetts General Hospital (MGH), Massachusetts Institute of Technology (MIT) and Harvard, Cambridge, MA, United States; ^2^ Department of Microbiology, Harvard Medical School, Boston, MA, United States

**Keywords:** SARS-CoV-2, coronavirus, immune imprinting, receptor binding domain (RBD), spike (S)

## Abstract

Since the emergence of SARS-CoV-2 (SARS-2), multiple vaccine candidates were developed and studied both preclinically and clinically. Nearly all are based on the SARS-2 spike glycoprotein or its receptor binding domain (RBD). Studies of these vaccine candidates have largely been in a SARS-2 naïve context. However, pre-existing immunity to SARS-2 acquired through infection or vaccination continues to increase. Evaluating future vaccine candidates in context of this pre-existing immunity is necessary to understand how immune responses are subsequently influenced. Here, we evaluated the serum and IgG^+^ B cell responses to the SARS-2 RBD in context of pre-existing immunity elicited by the full SARS-2 spike, and we compared this to boosting with the full SARS-2 spike. Boosting with the SARS-2 RBD resulted in increased reactivity to RBD epitopes, but both immunization regimens resulted in similarly broad neutralization across diverse sarbecoviruses. These findings may inform comparison among SARS-2 RBD-based vaccine candidates to currently approved spike-based candidates.

## Introduction

Since the emergence of SARS-CoV-2 (SARS-2), multiple vaccine candidates have been developed (1, 2). Several candidates use the full SARS-2 spike in various modalities (*e.g.*, mRNA, adenoviral vectors, recombinant protein) have received WHO and country-level regulatory approval; other vaccine candidates include only the SARS-2 receptor binding domain (RBD) (3–11). Several RBD-based vaccine candidates are advancing as potential pan-sarbecovirus vaccines and may eventually be used clinically (4–6, 12–14). If these RBD-based vaccine candidates are used, it is increasingly likely that many will already have pre-existing immunity to SARS-2 because of previous infection or immunization with currently approved vaccines (15). It is therefore necessary to evaluate the immunogenicity of the SARS-2 RBD in the context of SARS-2 spike imprinting.

The extent to which immune imprinting can bias antibody responses has previously been evaluated in the context of sequential viral infections and immunizations (16–18). These studies have predominantly focused on influenza, where imprinting has clinical relevance and often reduces seasonal vaccine efficacy (16–21). However, comparatively little is known about the impact of immune imprinting and the influence on immune responses following subsequent immunizations against SARS-2 (15). Previous infections with common cold-causing coronaviruses have been shown to bias the SARS-2 antibody response, and individuals with a history of prior SARS-1 infection generate antibody responses following SARS-2 vaccination that have a distinctly broad neutralization pattern (22, 23). Additionally, there is evidence that the SARS-2 variant with which an individual was initially infected can alter the imprinted immune response (24). Since RBD-based vaccine candidates may be used as boosting immunogens for potential pan-sarbecovirus immunity, evaluating the impact of boosting with this subunit is necessary.

In this study, we evaluated the serum and IgG^+^ B cell responses to boosting with the SARS-2 RBD in the context of pre-existing immunity elicited by the full SARS-2 spike, and we compared this to boosting with the full SARS-2 spike. While these two boosting immunogens resulted in similar serum antibody titers to multiple sarbecovirus RBD proteins, boosting with the full spike protein resulted in slightly broader neutralization against related coronaviruses. At the IgG^+^ B cell level, boosting with the SARS-2 RBD or spike resulted in B cell receptors targeting the RBD and non-RBD portions of the spike, respectively. Our data supports that boosting with RBD or full spike can offer broad sarbecovirus serum binding and neutralization, but that boosting with the RBD might be preferable in instances where it would be useful to bias memory towards RBD epitopes.

## Results

### Immunization regimens generate cross-reactive antibody responses

**Figure 1:**
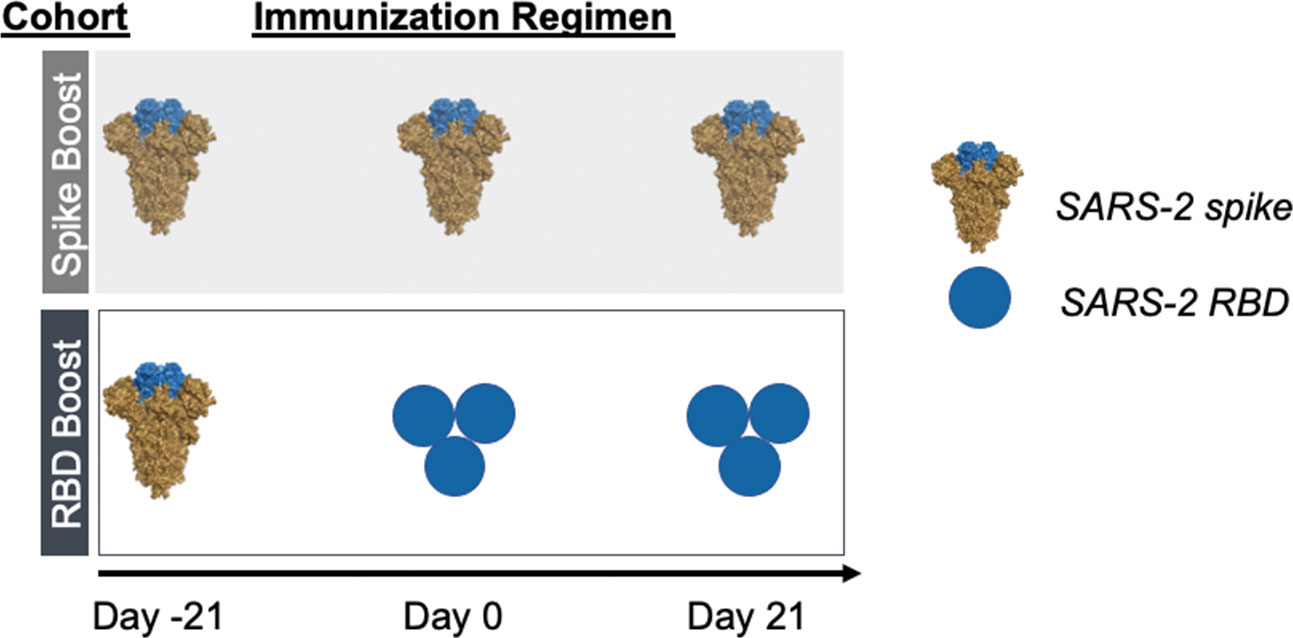
Immunization regimens. Two immunization cohorts were used for this study (n=5 mice per cohort). Mice were primed with SARS-2 2-proline- stabilized spike protein on day -21 and then boosted with either the same spike protein (“Spike Boost” cohort) or a trimeric SARS-2 RBD (“RBD Boost” cohort) at days 0 and 21.

As a surrogate of preexisting immunity to SARS-2, we primed our cohorts with recombinant stabilized SARS-2 spike protein (25, 26). Following this, mice were homologously boosted with recombinant SARS-2 spike (“Spike Boost” cohort) or SARS-2 RBD trimer (“RBD Boost” cohort) (**Figure 1**). We used a previously described hyperglycosylated, cysteine-stabilized GCN4 tag to trimerize the RBDs (12, 27) to improve overall immunogenicity relative to the monomeric RBD; this also ensured comparable avidity to the trimeric spike protein, which includes three copies of the RBD. All cohorts received 20 μg of recombinant protein adjuvanted with Sigma Adjuvant (28) at days -21, 0, and 21.

**Figure 2:**
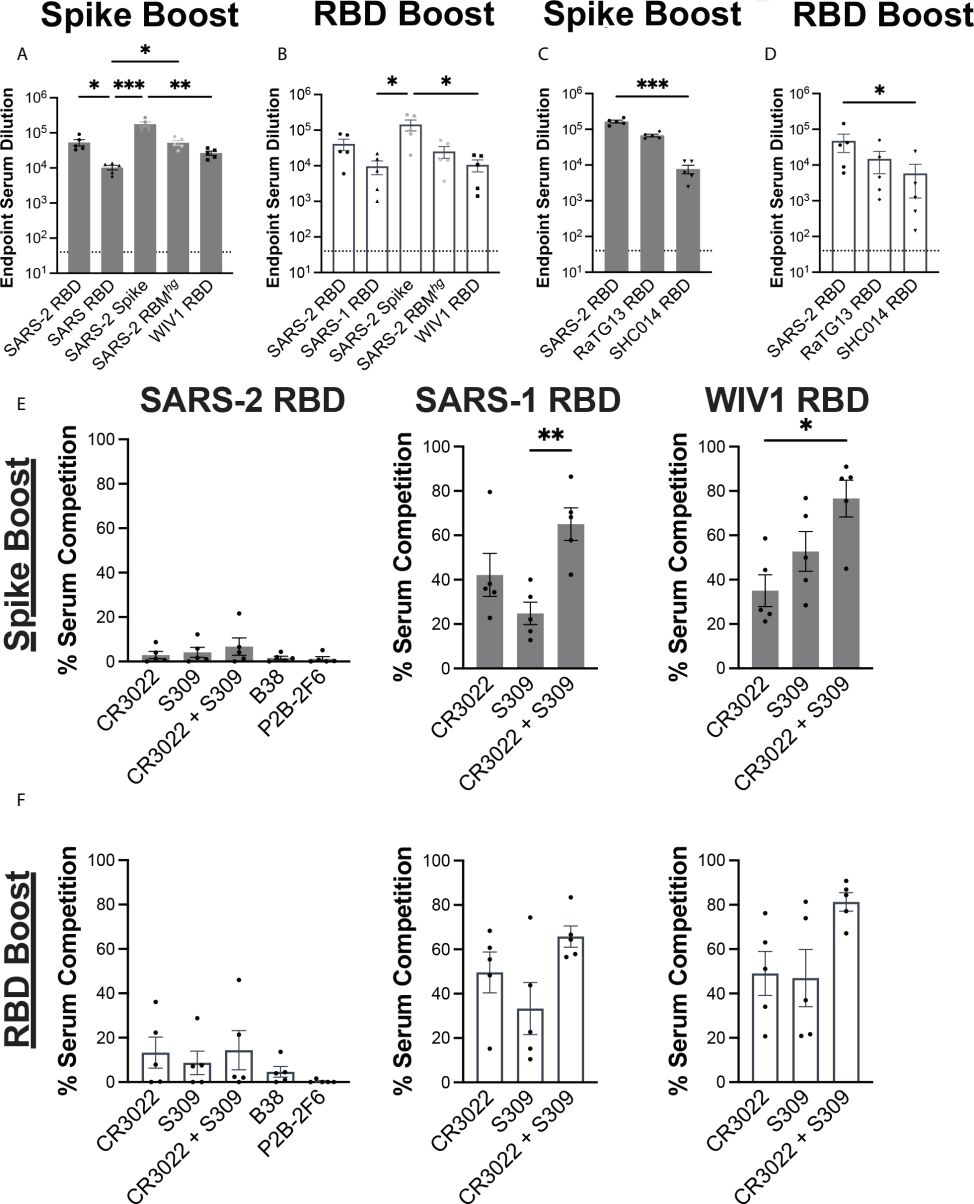
Serum response to immunization regimens. Serum collected at day 35 from the Spike Boost cohort **(A)** and the RBD boost cohort **(B)** was assayed in ELISA different coronavirus antigens. **(C, D)** Additional ELISAs were performed using day 35 serum samples against RaTG13 and SHC014 RBDs. **(E, F)** Percent of day 35 serum antibody binding lost in competition ELISAs compared to a no IgG control. SARS- 2, SARS-1, and WIV1 RBDs were used as coating antigens. Statistical significance across all panels was determined using Kruskal-Wallis test with post-hoc analysis using Dunn’s test corrected for multiple comparisons (*p < 0.05, **p < 0.01, ***p < 0.001). All pairwise comparisons were performed, and only statistically significant comparisons are indicated on the plots.

We evaluated the serum response against coronavirus-derived antigens using ELISAs, including SARS-2 spike, RBDs from SARS-2, SARS-1, WIV1, as well as a previously described SARS-2 RBM*^hg^* RBD with two engineered glycans at positions 475 and 501 that abrogate ACE2 engagement (**Figures 2A, B**) (12, 29). Both immunization regimens resulted in similar patterns of serum reactivity. Each cohort had a significant decrease in reactivity against the SARS-1 and WIV1 RBDs relative to the SARS-2 spike, with around a 10-fold difference. Both the Spike Boost cohort and the RBD Boost cohort had the highest ELISA titers against the full SARS-2 spike protein. The Spike Boost cohort also showed a significant decrease in reactivity against the SARS-1 RBD in comparison to both the SARS-2 RBD and the SARS-2 RBM*^hg^* RBD.

We further tested whether the sera from each cohort could cross-react with additional sarbeocoviruses, RaTG13 and SHC014 (**Figures 2C, D**). While both cohorts showed a significant decrease in reactivity against the SHC014 RBD relative to the SARS-2 RBD, the magnitude of this difference was greater in the Spike Boost cohort. These data suggest that boosting with either the SARS-2 spike or RBD confers broad reactivity to related sarbecoviruses.

### Serum responses map onto conserved epitopes

We next evaluated whether the serum response was directed towards cross-reactive, and potentially broadly neutralizing RBD epitopes. Conservation across SARS-2, SARS-1, and WIV1 RBDs primarily occurs outside of the ACE2 receptor binding motif. Indeed, the previously characterized CR3022 and S309 antibodies have footprints that together cover much of this conserved region, with epitope buried surface area (BSA) of 917 Å^2^ and 795 Å^2^, respectively compared to 869 Å^2^ for ACE2 (30–32). We performed serum competition by incubating RBD-coated ELISA plates with IgGs B38, P2B-2F6, CR3022, and S309, representing each of the four previously defined “classes” of SARS-2 RBD epitopes (33). We then assessed binding of mouse serum IgG to determine the extent of serum competition with each monoclonal antibody (**Figures 2E, F**). In both cohorts, competition with both CR3022 and S309 reduced serum titers against the SARS-1 and WIV1-1 RBDs, though this difference was only statistically significant in the Spike Boost cohort. Neither the Spike Boost cohort nor the RBD Boost cohort showed a significant reduction in serum titers against the SARS-2 RBD in competition with any of the antibodies, indicating that the serum antibody response is not focused predominantly to these epitopes.

### Expanded IgG^+^ B cell populations target cross-reactive receptor binding domain epitopes

**Figure 3:**
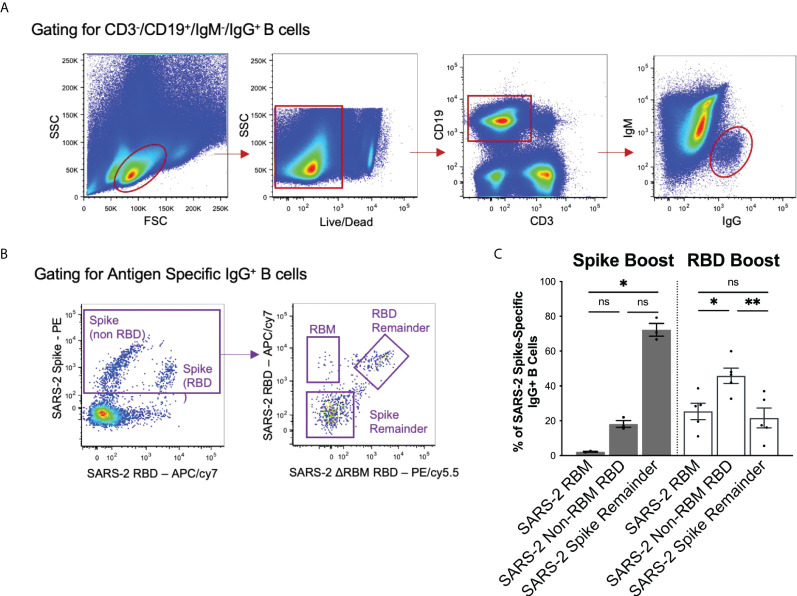
Flow cytometry gating scheme and results. **(A)** Gating strategy to select for CD3-/CD19+/IgM-/IgG^+^ cells to isolate the memory B cell population. **(B)** Antigen specific memory IgG responses were identified using a combination of SARS-2 spike, SARS-2 RBD, and SARS-2 RBM*^hg^* RBD flow hooks. SARS-2 spike-directed B cell responses were further separated by reactivity to into the RBM, RBD remainder (excluding RBM epitopes), and spike remainder (excluding RBM and RBD epitopes). **(C)** Spike-directed responses were binned into RBM, RBD remainder (excluding RBM epitopes), and spike remainder (excluding RBD and RBM epitopes) populations using relevant probes in flow cytometry. Data is shown as a percentage of total spike-specific IgG^+^ B-cells. Statistical significance was determined using Kruskal-Wallis test with posthoc analysis using Dunn’s test corrected for multiple comparisons (*p < 0.05, **p < 0.01); ns, not significant.

To compare the observed sera responses, we measured the amount of antigen-specific IgG^+^ B cells expanded by the Spike Boost and RBD Boost immunization regimens. We used the SARS-2 RBM*^hg^* RBD construct with the two additional glycans on the RBM to bin SARS-2 spike-directed B cells into 3 populations: those that bound RBM epitopes; those that bound the non-RBM epitopes on the RBD; and those that bound the “remainder” of the spike protein (**Figures 3A, B**). In the RBD Boost cohort, the proportion of B cells specific for the non-RBM portion of the RBD was significantly greater than the proportion of B cells directed towards the RBM or the remainder of the SARS-2 spike (**Figure 3C**). In the Spike Boost cohort, most SARS-2 spike-directed B cells bound to the non-RBD regions on the SARS-2 spike. This indicates that boosting with the SARS-2 RBD, rather than the full SARS-2 spike, redirects the IgG^+^ B cell response towards RBD epitopes and away from epitopes in the SARS-2 spike that fall outside of the RBD.

### Elicited immune response is cross-neutralizing

**Figure 4:**
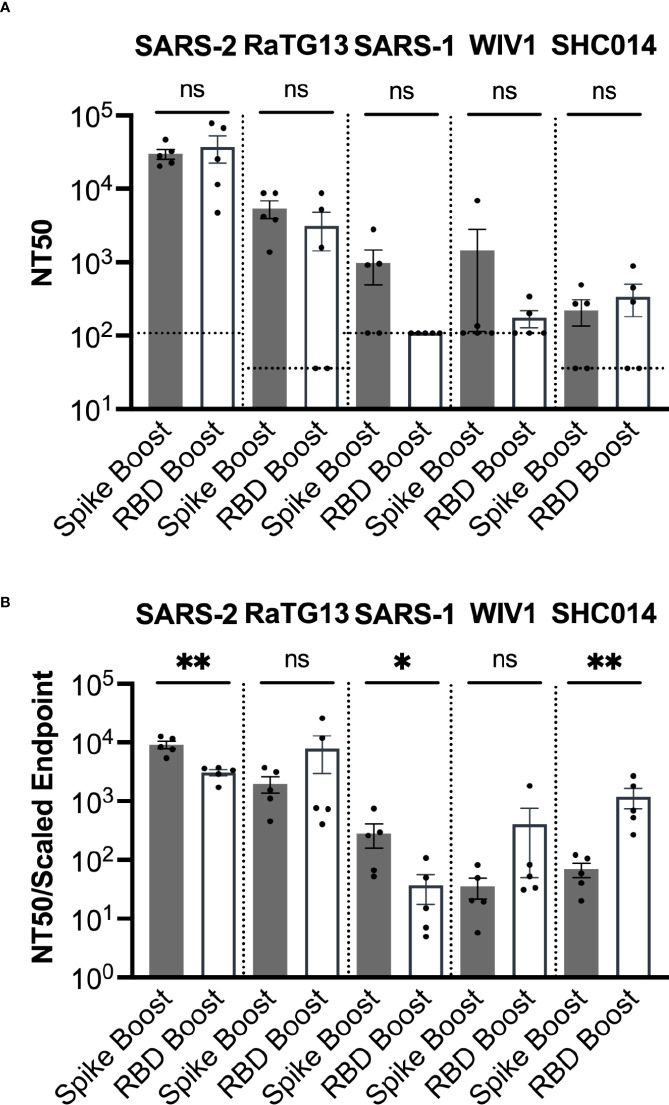
Sarbecovirus neutralization. **(A)** Neutralization was assayed across a range of sarbecoviruses, ordered here from left to right based on genetic similarity of the spike gene to SARS-2. **(B)** Neutralization potency was computed by dividing the NT50 value for each sample by the adjusted serum ELISA endpoint titer for the corresponding sarbecovirus RBD. Comparisons across both panels were performed using the Mann-Whitney U test (*p < 0.05, **p < 0.01); ns, not significant.

We next determined serum neutralization profiles from each cohort against SARS-2, as well as the related sarbecoviruses SARS-1, WIV1, RaTG13, and SHC014. Several of these viruses are of potential pandemic concern and have been detected in bats but not yet in humans (34, 35). We performed pseudovirus neutralization assays and obtained NT50 values (36) (**Figure 4A**). However, we note that most serum samples for which NT50 values could not be determined still had some weak neutralizing activity (16 out of 19 samples).

NT50 values for both cohorts were highest against SARS-2, which was consistent with the high titers of antibodies against the SARS-2 RBD and spike proteins (**Figure 4A**). Sera had the second highest NT50 values against the closely related RaTG13. While some neutralization was detected against the more distantly related sarbecoviruses SARS-1, WIV1, and SHC014, these NT50 values (~10^2^ – 10^3^) were considerably less than those corresponding to SARS-2 (~10^5^) and RaTG13 (~10^4^).

We also obtained the neutralizing potency response, calculated by dividing the NT50 value measured in ELISA titer against the RBD of the same virus (**Figure 4B**). The Spike Boost cohort had significantly more potent neutralization against SARS-2 and SARS-1, while the RBD Boost cohort had significantly more potent neutralization against SHC014. The RBD Boost cohort also had more potent neutralization of RaTG13 and WIV1, but these differences were not statistically significant. These data suggest that boosting with either the SARS-2 spike or the RBD results in similarly broad cross-neutralization of related sarbecoviruses.

## Discussion

In this study, we compared the serum and IgG^+^ B cell responses to boosting with the full SARS-2 spike or RBD in the context of pre-existing immunity. We found that the Spike Boost cohort had improved neutralization breadth against related coronaviruses, though the RBD Boost cohort had a more potent neutralizing response against some of the coronaviruses tested. This indicates differences in the nature of the antibody response elicited by the two boosting regimens, as reflected in the increased percentage of RBD-specific IgG^+^ B cells elicited in the RBD Boost cohort.

As prior vaccination or infection with SARS-2 is increasing, the response elicited by candidate immunogens in a naïve context is less relevant (15). Immune imprinting *via* prior infection or vaccination significantly biases the antibody response upon subsequent exposures to different strains of the same virus; this phenomenon is especially well-characterized in the context of influenza (16–18). The emergence of the Omicron variant demonstrated that additional boosting with wildtype SARS-2 spike can enhance protection against antigenically divergent variants; further boosting immunizations may be required as additional variants continue to evolve or if additional sarbecoviruses enter the human population (36–44). Furthermore, numerous candidate immunogens currently in development as potential pan-sarbecovirus vaccines use the SARS-2 RBD, while others use the full spike (3, 5–11, 13, 26, 45–48). Consequently, the impact of boosting with the SARS-2 RBD rather than the full spike in the context of pre-existing immunity merits evaluation. It is likely that different exposure histories (e.g., infection, vaccination) within an individual could uniquely shape responses to subsequent SARS-2 boosting; further studies are needed to systematically characterize potential response patterns, though these studies are likely to be limited in the murine model.

This study characterized the immunological profiles after boosting with either SARS-2 RBD or spike. We observed a bias towards the full SARS-2 spike in both immune responses, as evidenced by the fact that ELISA titers were highest against this protein. Pseudotyped lentiviruses for neutralization assays allowed us to compare the breadth and potency of neutralization elicited by each immunization regimen, and flow cytometry defined the specificity of the resulting IgG^+^ B cell response. Importantly, the SARS-2 RBD immunogen was trimerized to match the valency of the spike protein. However, further multimerization of both constructs (*e.g.*, nanoparticles) was previously shown and may improve the immunogenicity of one or both immunogens beyond levels observed here (5–10, 49).

While this study does not characterize the relative protection resulting from each boosting regimen, significant evidence already exists that both spike-based and RBD-based protein immunizations provide protection (3, 26, 42, 47, 50–53). In combination with the findings of this study, these data support using either RBD-based or spike-based immunogens for subsequent boosts to confer broad neutralization against SARS-2 and related sarbecoviruses. However, boosting with RBD-based immunogens may be advantageous if directing memory response towards RBD epitopes is preferred. Many potently neutralizing SARS-2 therapeutic antibodies target RBD epitopes, including antibodies that can neutralize other sarbecoviruses and SARS-2 variants (33, 54–57); additionally, spike contains several highly conserved epitopes outside the RBD, including neutralizing epitopes in the stem helix and fusion peptide regions (58–60). Preferred epitopes targeted by future sarbecovirus vaccines should take into consideration whether to boost with RBD or spike-based immunogens.

## Methods

### Spike and receptor binding domain expression and purification

Receptor binding domains (RBDs) were designed based on the following sequences: SARS-2 RBD (Genbank MN975262.1), SARS-1 RBD (Genbank ABD72970.1), WIV1 RBD (Genbank AGZ48828.1), RaTG13 RBD (Genbank QHR63300.2), SHC014 RBD (Genbank QJE50589.1). Constructs were codon optimized by Integrated DNA Technologies, cloned into pVRC, and sequence confirmed by Genewiz. The spike plasmid was obtained from Dr. Jason McLellan at the University of Texas, Austin. It contained a Foldon trimerization domain as well as C-terminal HRV 3C-cleavable 6xHis and 2xStrep II tags. Proteins were expressed in Expi293F cells (ThermoFisher) using Expifectamine transfection reagents according to the manufacturer’s protocols. All proteins included a C-terminal HRV 3C-cleavable 8xHis tag to facilitate purification. Monomeric RBD proteins also contained SBP tags, while homotrimeric constructs contained a previously published hyperglycosylated GCN4 tag with two additional C-terminal cystines (27). A linker with the sequence GASSGSG separated each RBD from the hyperglycosylated GCN4 tag.

Transfections were harvested after 5 days and clarified *via* centrifugation. Cell supernatants were passaged over Cobalt-TALON resin (Takara) for immobilized metal affinity chromatography *via* the 8xHis tag. After elution, proteins were passed over a Superdex 200 Increase 10/300 GL (GE Healthcare) size exclusion column in PBS (Corning). Prior to immunization, 8xHis tags were cleaved using HRV 3C protease (ThermoScientific). Cleaved protein was repurified using Cobalt-TALON resin in order to remove the protease, cleaved tag, and any uncleaved protein.

### IgG expression and purification

Genes for the variable domains of the heavy and light chains were codon optimized by Integrated DNA Technologies and cloned into pVRC constructs containing the respective constant domains as previously described (61, 62). Heavy-chain IgG constructs contained HRV 3C-cleavable 8xHis and SBP tags. Transfections and purifications were performed according to the same protocols used for the RBDs and homotrimers.

### Immunizations

C57BL/6 mice (Jackson Laboratory) received 20 μg of protein adjuvanted with 50% w/v Sigma Adjuvant System in 100 μL of inoculum (28). All immunizations were administered through the intraperitoneal route. Mice were primed (day -21) and received boosting immunizations at day 0 and day 21. Serum samples were collected on day 35 for characterization, with flow cytometry occurring between days 35 and 42. In this study, female mice aged 6-10 weeks were used. All experiments were conducted with institutional IACUC approval (MGH protocol 2014N000252).

### Flow cytometry

Spleens were isolated from mice and single cell suspensions were generated by straining through a 70 μm cell strainer. Red blood cells were removed by treating with ACK lysis buffer and washed with PBS. Single cell suspensions were first stained with Aqua Live/Dead amine-reactive dye (0.025 mg/mL) before applying the following B and T cell staining panel using the staining approach described previously (28, 63). This included the following mouse-specific antibodies: CD3-BV786 (BioLegend), CD19-BV421 (BioLegend), IgM-BV605 (BioLegend), IgG-PerCP/Cy5.5 (BioLegend).

Streptavidin-conjugated fluorophores were used to label the SBP-tagged proteins as probes for flow cytometry. For the cohort that received the SARS-2 RBD trimer boost, the following probes were generated: SARS-CoV-2 RBD-APC/Cy7 (streptavidin-APC/Cy7 from BioLegend), SARS-CoV-2 spike-StreptTactin PE (StrepTactin PE from IBA Lifesciences), SARS-CoV-2 RBH*^g^* RBD-PE/Cy5.5 (streptavidin-PE/Cy5.5 from BioLegend). For the cohort that received three SARS-CoV-2 spike immunizations, the following probes were generated: SARS-CoV-2 RBD-APC/Cy7 (streptavidin-APC/Cy7 from BioLegend), SARS-CoV-2 spike-StreptTactin PE (StrepTactin PE from IBA Lifesciences), SARS-CoV-2 RBH*^g^*-APC (streptavidin-APC from BioLegend). Conjugations were performed as previously described (64). Briefly, fluorescent streptavidin conjugates were added in 5 increments with 20 minutes of incubation with rotation at 4°C in between to achieve a final molar ratio of probe to streptavidin valency of 1:1. The final conjugated probe concentration was 0.1 μg/mL. Flow cytometry was performed on a BD FACSAria Fusion cytometer (BD Biosciences). Analysis of the resultant FCS files was conducted using FlowJo (version 10).

### Serum ELISAs

Serum ELISAs were performed by coating Corning 96-well clear flat bottom high bind microplates with 100 μL of protein at 5 μg/mL in PBS. Plates were incubated overnight at 4°C. Coating solution was removed, and plates were blocked using 1% BSA in PBS with 1% Tween for 60 minutes at room temperature. Blocking solution was removed. Sera were diluted 1:40 in PBS, and 5-fold serial dilution was performed. CR3022 IgG at a starting dilution of 5 μg/mL with 5-fold serial dilution was used as a positive control. 40 μL of primary antibody solution was applied to each well. Primary incubation occurred for 90 minutes at room temperature. Plates were then washed three times with PBS-Tween. HRP-conjugated rabbit anti-mouse IgG antibody (Abcam) at a concentration of 1:20,000 in PBS and a volume of 150 μL was used as a secondary antibody. Secondary incubation occurred for 60 minutes at room temperature. Plates were then washed three times with PBS-Tween. 1xABTS development solution (ThermoFisher) was applied as outlined in the manufacturer’s recommendations. Development was stopped after 30 minutes with a 1% SDS solution. Plates were read at 405 nm using a SectraMax iD3 plate reader (Molecular Devices).

### Competition ELISAs

Competition ELISAs were performed using a similar protocol to serum ELISAs. The primary incubation consisted of 40 μL of the relevant IgG at 1 μM. Incubation occurred at room temperature for 60 minutes. Mouse sera were then spiked in at a final concentration in the linear range of the serum ELISA titration curve (1:800 for the cohort that received three SARS-CoV-2 RBD monomer immunizations, 1:12,800 for all other cohorts). Plates were incubated at room temperature for an additional 60 minutes. The primary solution was removed, and plates were washed three times using PBS-Tween. HRP-conjugated goat anti-mouse IgG, human/bovine/horse SP ads antibody (Southern Biotech) was applied at a concentration of 1:4000 and a volume of 150 μL as a secondary antibody. Plates were then incubated, washed, and developed using the same procedure as the serum ELISAs.

### Pseudovirus neutralization assay

Serum neutralization against SARS-CoV-2, SARS-CoV, and WIV1-CoV was assessed using lentiviral particles pseudotyped with the respective spike proteins as previously described (36). Lentiviral particles were produced *via* transient transfection of 293T cells. The titers of viral supernatants were determined *via* flow cytometry on 293T-ACE2 cells (65) and *via* the HIV-1 p24^CA^ antigen capture assay (Leidos Biomedical Research, Inc.). Assays were performed in 384-well plates (Grenier) using a Fluent Automated Workstation (Tecan). For mouse sera, samples were initially diluted 1:9, with subsequent serial 3-fold dilutions. Serum sample volume in each well was 20 μL, and 20 μL of pseudovirus containing 125 infectious units was added. The combination was incubated for 60 minutes at room temperature. Afterwards, 10,000 293T-ACE2 cells (65) in 20 μL of media containing 15 μg/mL polybrene was added. The plates were then incubated at 37°C for 60-72 hours.

A previously described assay buffer was used to lyse the cells (66). A Spectramax L luminometer (Molecular Devices) was used to quantify luciferase expression. Percent neutralization at each serum concentration was determined by subtracting background luminescence from cells only sample wells, then dividing by luminescence of wells with only virus and cells. GraphPad Prism was used to fit nonlinear regressions to the data, which allowed IC_50_ values to be calculated using the interpolated 50% inhibitory concentration. IC_50_ values were calculated for all samples with a neutralization value of at least 80% at the highest serum concentration.

## Data availability statement

The original contributions presented in the study are included in the article/supplementary material. Further inquiries can be directed to the corresponding authors.

## Ethics statement

The animal study was reviewed and approved by Partners Institutional Biosafety Committee for institutional IACUC approval (MGH protocol 2014N000252).

## Author contributions

Conceptualization, BH and AS. Methodology, BH, MS, EL, AB, DL, and AS. Investigation, BH, MS, EL, and JF. Writing – original draft, BH and AS. Writing – review and editing, all authors. Funding acquisition, AB, DL, and AS. Supervision, AB, DL, and AS. All authors contributed to the article and approved the submitted version.

## Funding

We acknowledge funding from NIH R01s AI146779 (AS), AI124378, AI137057 and AI153098 (DL), and a Massachusetts Consortium on Pathogenesis Readiness (MassCPR) grant (AS), training grants: NIGMS T32 GM007753 (BH), T32 AI007245 (JF), F31 Al138368 (MS), F30 AI160908 (BH). AB is supported by the National Institutes for Drug Abuse (NIDA) Avenir New Innovator Award DP2DA040254, the MGH Transformative Scholars Program as well as funding from the Charles H. Hood Foundation (AB). This independent research was supported by the Gilead Sciences Research Scholars Program in HIV (AB).

## Acknowledgments

We thank members of the Schmidt Laboratory for helpful discussions. We thank Dr. Jason McLellan from University of Texas, Austin for the spike plasmid. We thank Nir Hacohen and Michael Farzan for the kind gift of the ACE2 expressing 293T cells.

## Conflict of interest

The authors declare that the research was conducted in the absence of any commercial or financial relationships that could be construed as a potential conflict of interest.

## Publisher’s note

All claims expressed in this article are solely those of the authors and do not necessarily represent those of their affiliated organizations, or those of the publisher, the editors and the reviewers. Any product that may be evaluated in this article, or claim that may be made by its manufacturer, is not guaranteed or endorsed by the publisher.
